# Neonatal blood lead concentration predicts medium term lead-related outcomes in children ≤5 years old with congenital lead poisoning: A retrospective cohort study in Northern Nigeria

**DOI:** 10.1371/journal.pgph.0001644

**Published:** 2023-03-29

**Authors:** Natalie Thurtle, Katharine A. Kirby, Jane Greig, Karla Bil, Paul I. Dargan, Godwin N. Ntadom, Nicholas A. Buckley

**Affiliations:** 1 Médecins Sans Frontières, Operational Centre Amsterdam, Amsterdam, Holland; 2 Department of Pharmacology, University of Sydney, Sydney, NSW Australia; 3 Médecins Sans Frontières, Manson Unit, London, United Kingdom; 4 Clinical Toxicology, Guy’s and St Thomas’ NHS Foundation Trust, London, United Kingdom; 5 Faculty of Life Sciences and Medicine, King’s College London, London, United Kingdom; 6 Federal Ministry of Health, Abuja, Nigeria; 7 Nigerian Field Epidemiology and Laboratory Training Programme (NFELTP), Abuja, Nigeria; University at Buffalo, UNITED STATES

## Abstract

Mother-to-child-transmission of lead via the placenta is known to result in congenital lead toxicity. Between 2010 and 2021, Médecins Sans Frontières and other stakeholders responded to a severe lead poisoning outbreak related to artisanal gold mining in Northern Nigeria. Extensive environmental remediation occurred following outbreak identification; source control efforts are ongoing within the community. We aimed to describe the prevalence of congenital lead poisoning in this cohort and analyse the association between neonatal blood lead concentration (BLC) and medium-term lead-related outcomes during the study period. Children enrolled in the lead poisoning programme between July 2010 and 25 January 2018 who had a screening BLC at ≤4 weeks of age were included. For time-to-event analysis, medium-term outcomes were classified as lead-related (death from lead encephalopathy, and/or met chelation threshold) and non-lead-related (non-lead-related death, on programme no chelation, exit from programme without chelation). Cox regression analysis and ROC analysis were performed. 1468 children were included. All-cause mortality 2.3%; geometric mean neonatal BLC 13.7 μg/dL; ‘lead-related death or treatment’ 19.3%. For every doubling in neonatal BLC, there was an almost 8-fold increase in adjusted hazard ratio (HR) for the composite lead-related outcome (p<0.001). A neonatal BLC ≥ 15.0 μg/dL had 95% sensitivity for identifying children who went on to have the composite outcome (with specificity 67%; positive likelihood ratio 2.86). Congenital lead poisoning predicts ongoing exposure in this population, even after environmental remediation. This suggests a complex, early, multidisciplinary approach to source control and exposure management is required when elevated neonatal BLC is observed in lead poisoning clusters in low-and-middle-income contexts.

## Introduction

Mother-to-child-transmission of lead via the placenta results in congenital lead poisoning. Foetal exposure to lead is known to impact neurodevelopment [1] and be linked to prematurity and low birth weight [2]. Post-natal childhood exposure to lead has been shown to have far-reaching consequences on multiple body systems, even at levels previously thought to be ‘safe’ [3].

Médecins Sans Frontières (MSF) and other stakeholders responded to a large acute severe lead poisoning outbreak in Zamfara state, Northern Nigeria between 2010 and 2021. An estimated 400 children <5 years old died in the acute emergency, many before the outbreak was identified and before a response could be mounted [4–6]. Subsequently over 7500 children were screened for lead poisoning, with over 35,000 courses of the oral chelating agent dimercaptosuccinic acid (DMSA) administered [7,8]. BLC data and a limited clinical dataset were collected and recorded operationally to support clinical management of children enrolled in the programme and to inform environmental responses and interventions.

The source of exposure was primarily soil contamination from lead dust generated from artisanal gold mining [9,10]. Plumlee et al found that sweep and soil samples from the two villages first identified with child fatalities from lead poisoning contained particles of the same lead minerals as vein ores from the region, confirming ore processing as the source of contamination. Food staples such as poultry, cereal grains and legumes were contaminated from the same source and likely contributed to lead ingestion [11–13]. Breast milk studies elsewhere have demonstrated milk to contain ≤3% of maternal blood lead concentration (BLC) [14] and it is therefore not considered a significant causal contributor to severe lead poisoning. Increasing BLC postnatally are likely related to ongoing environmental exposure.

Large-scale environmental remediation was carried out in stages over the first years of the outbreak by TerraGraphics International Foundation and Zamfara State Government Ministry of Environment [9]. A follow up programme was put in place for repeat remediation of areas re-contaminated following remediation. Work to support the community with safer mining initiatives was ongoing as of end of 2022 [15], however modulation of behaviours around artisanal mining is challenging. Unsafe Mining Practices include but are not limited to grinding and processing of ore in domestic compounds, clothing, ore and utensils from artisanal mining activities being stored in the domestic compound where children can access them, children sharing food close to areas where ore is being ground, no hand washing facilities at processing sites.

As such, even though living areas have been remediated, we hypothesized that children with higher neonatal BLC, i.e., those whose mothers have higher BLC contributing to placental transmission, may be more at risk of post-partum exposure due to environmental associations, and therefore be more likely to have an outcome related to severe lead poisoning.

Understanding whether neonatal BLC is a reliable predictor of outcome in an acute lead poisoning outbreak is potentially useful for pragmatic resource management in low resource settings. Distinguishing between those that need priority source control and mandatory follow up to prevent serious morbidity and mortality from lead poisoning versus those that can be discharged (in the context of robust source control measures) could be an important tool for those managing outbreaks in difficult contexts.

Source control with regards to lead contamination from artisanal mining is a myriad of activities aimed at reducing production and distribution of lead dust and cleaning up or remediating areas shown to be contaminated. These measures include but are not limited to: moving grinding and processing sites far away from domestic areas, provision of hand washing facilities and personal protective equipment, leaving clothing, equipment and ore at the processing site, wet milling, provision of information and empowerment of communities to find solutions to minimise lead contamination.

The aims of this study are to describe the prevalence of congenital lead poisoning in an acute severe outbreak and to describe and analyse the association between neonatal BLC and medium-term lead-related outcomes during the study period. This manuscript uses this operationally collected data to focus on children with pre-natal lead exposure defined as a documented raised neonatal BLC.

## Methods

This report adheres to the STROBE checklist for observational studies.

### Ethics statement

This work met the standards set by the independent MSF Ethics Review Board for retrospective analyses of routinely collected programmatic data [16]. These standards include, but are not limited to, assurances of confidentiality, involvement of local partners, and minimal risk of harm to patients. Caregivers were informed that routine data were being collected for clinical care and could be de-identified and used for programme monitoring and increasing knowledge about lead poisoning. Informed consent was not sought for publication of this fully de-identified aggregate data that was not collected for research purposes. Coded identification numbers were used, and personal identifiers and all unnecessary data were removed from the dataset prior to analysis. Review of de-identified, routinely collected programmatic data did not constitute research under the Nigerian National Health Research Ethics Committee published guidelines and therefore formal ethical approval was not sought [17].

### Study design

This was an observational cohort study analysing data collected solely for the purpose of providing treatment to children with lead poisoning and not for research purposes.

### Sampling

Venepuncture was performed by trained medical staff utilising soap and water to eliminate lead dust from the skin as a potential contaminant. BLC testing was performed by trained lab technicians using the point-of-care analyser Lead Care II (Magellan) (LCII) according to manufacturer protocols. Lead Care II utilizes anodic stripping voltammetry (ASV). Lead Care II has a lower limit of detection of 3.3 μg/dL and an upper limit of detection of 65 μg/dL [18]. It is noted that Lead Care II blood lead test kits were the subject of an FDA recall in 2021 due to a risk of falsely low results. Monthly quality control monitoring for BLC results obtained using LCII was conducted with Inductively Coupled Plasma Mass Spectrometry at the Centers for Disease Control, Atlanta, USA. Point-of-care LCII values were on average 4.0 μg/dL lower than ICPMS values (limits of agreement [19] {two standard deviations] -19.7 to +11.7 μg/dL).

All BLC <3.3 μg/dL were recorded as 3.3 μg/dL for analysis. For samples with BLC >65 μg/dL, a blood dilution method utilising donor blood with BLC <3.3 μg/dL was developed and implemented [20].

### Inclusion criteria

**Fig 1:**
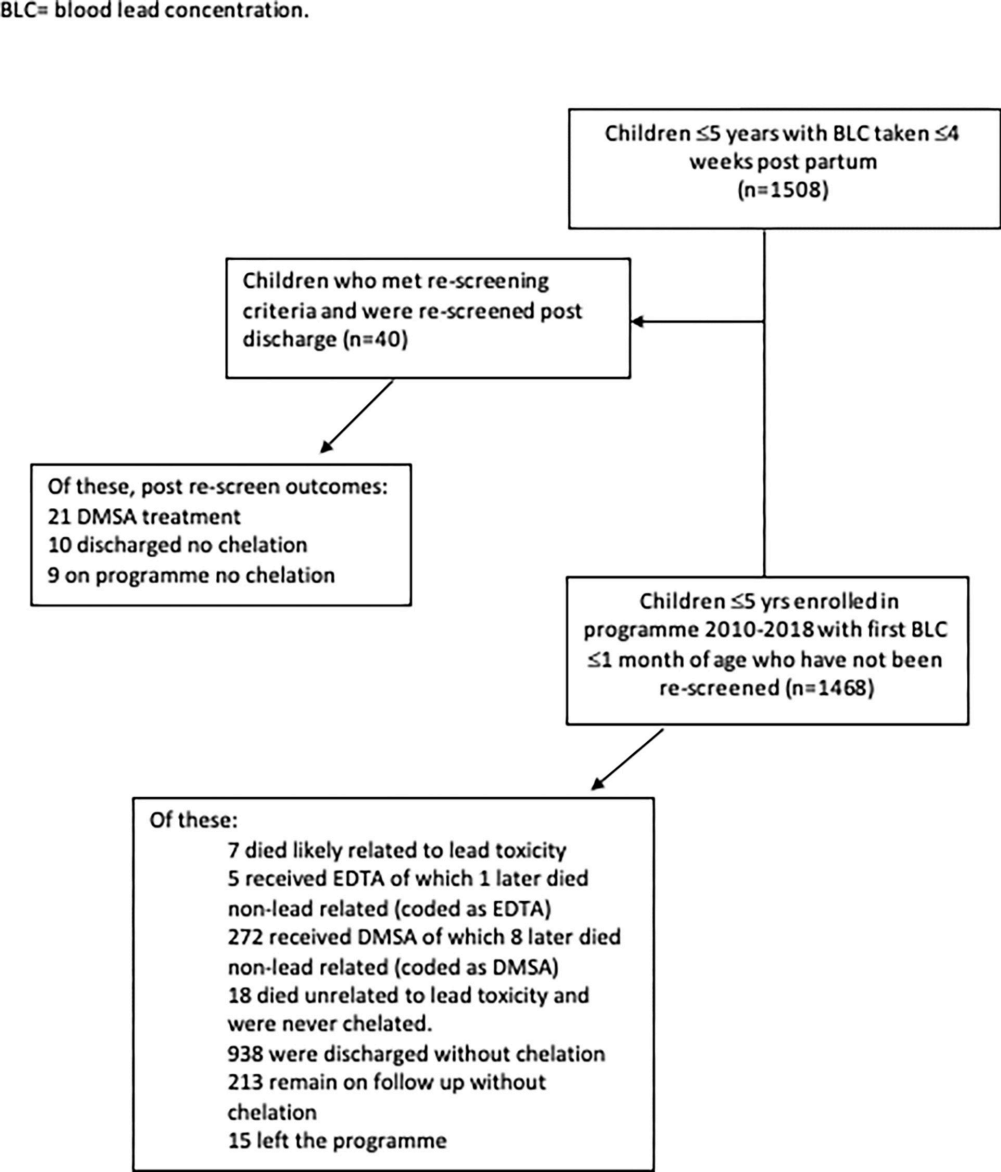
Flow chart of inclusion and exclusion of children. BLC = blood lead concentration.

We included 1468 children enrolled in the Zamfara lead poisoning programme between July 2010 and 25 January 2018 who had a screening BLC at ≤4 weeks of age (Fig 1, flowchart). Children were eligible for enrolment in the treatment programme if they were < = 4 years of age at initial screening and lived in one of 8 villages in Zamfara state identified as contaminated with lead or were living elsewhere but were brought for screening. A specific sample size was not calculated and all children who met the inclusion criteria were evaluated.

### Data collection

Other variables collected were sex, age at the time of initial screening (1–4 weeks), year of initial screening, and village of usual residence (one of 8 villages or ‘other’), as the villages were known to have different exposure profiles.

The mean BLC of a group of breast-feeding mothers tested early in the outbreak (June to September 2010) was also reported to provide context of maternal BLC during the early stages of the outbreak. The duration of time post-partum at time of BLC testing was not recorded and no maternal-child matched BLC are available.

Data were collected and input into a custom-designed database (Microsoft Excel 2007) between 2010 and 2018 by the operational team during clinical activities. Data was extracted and input into a Microsoft Excel spreadsheet for geometric mean and standard deviation calculations and into Stata (StataCorp. 2015. *Stata Statistical Software*: *Release 14*. College Station, TX: StataCorp LP) for further analysis. The Forest plot was created in GraphPad Prism.

### Data analysis

Included children were assigned one of the following 7 medium-term outcomes as of 25^th^ January 2018. 1) ‘EDTA’–defined as child met protocol criteria for requiring a course of calcium disodium edetate (EDTA) (BLC ≥45 μg/dL on any blood test AND meets the definition of severe encephalopathy–active seizures on presentation, status epilepticus or an AVPU (Alert, responds to Voice, responds to Pain, Unresponsive) [21] score of P (responsive only to pain) or U (unresponsive)); 2) ‘DMSA’–outcome defined as child met protocol criteria for requiring a course of DMSA (BLC ≥45 μg/dL) on any BLC; 3) ‘Died Not-Lead-Related’–outcome defined as child who died during the included period before having a lead related outcome and met the following criteria: last BLC ≤45 μg/dL within 3 months of death AND/OR clearly identified non-lead related cause of death regardless of BLC (e.g. trauma); 4) ‘Died Lead-related’–outcome defined as child who died and met one or more of the following criteria: last BLC ≥80 μg/dL and no clear non-lead related cause (fall etc), died with clinical features consistent with lead encephalopathy and last BLC 45–79 μg/dL, last BLC ≥3 months ago and cause of death unclear (included as cause of death unknown and therefore lead cannot be excluded); 5) ‘Left Programme No Chelation’–outcome defined as child left the programme against medical advice (defaulted) prior to meeting another outcome; 6) ‘On Programme No Chelation’–outcome defined as child remained on monitoring programme as of 25^th^ Jan 2018 without meeting any other outcome definition; 7) ‘Discharged No Chelation’–outcome defined as child met MSF protocol discharge criteria at time of discharge (S1 Annex, details of protocol development in Thurtle et al^3^).

Children who had outcomes 1 (EDTA) or 2 (DMSA) who then went on to die a death unrelated to lead were analysed as outcome 1 or 2 (lead related), though deaths were captured and reported in the all-cause mortality as non-lead related.

In 2017 a process began whereby children ≤3yrs living in a compound with children who had been identified with significant ongoing lead exposure either by having a BLC ≥100 μg/dL or a rise of ≥50% between two BLC were offered re-screening if they had been discharged from the programme, as it became clear that ongoing exposure was likely. Patients who had an outcome of ‘discharged no chelation’ who met re-screening criteria and were re-screened during the study period were excluded from the study (40 children). This was because they left the programme with an outcome and then re-entered later which would have complicated time-to-event (TTE) analysis. If children had already met a ‘Lead-related death or treatment’ outcome (DMSA, EDTA, death related to lead poisoning) prior to being discharged they were not excluded. The outcome of these 40 children after their re-admission to the programme is detailed in Fig 1, though they are excluded from the analysis.

Log base 2 transformation of BLC was performed to correct for positive skew and geometric means were calculated. Cox regression analysis was performed using Stata to evaluate TTE from neonatal BLC to outcome. As the dataset is observational and retrospective, explanatory variables for the Cox model were included if they were collected, available and met plausibility for association. Namely: BLC, sex, age in weeks at screening, village and year screened. Screening years 2011–2012 and 2017–2018 were combined as 2011 and 2018 contained a very small number of children. All covariates were included in the adjustment. The largest category was used as the reference for all variables. Test of proportional hazards assumption was completed on the Cox model and showed no violations. Villages with n≤50 children were combined for analysis into ‘other’. ROC analysis was performed.

For the purposes of TTE analysis, outcomes were dichotomised into composite outcomes of ‘Lead-related death or treatment’ or ‘No lead-related death or treatment’, with the former being classified as the failure outcome. ‘No lead-related death or treatment’ included ‘left programme no chelation’, ‘on programme no chelation’, ‘discharged no chelation’ and ‘death not lead-related’. ‘No lead-related death or treatment’ outcomes were all treated as non-informative censored.

Four Kaplan-Meier curves were constructed for time in months to composite binary outcome of ‘No lead-related death or treatment’ and ‘Lead-related death or treatment’ for BLC quartiles (3.3–8.7 mcg/dL, 8.8–13.7 mcg/dL, 13.8–23.1 mcg/dL and 23.2–105 mcg/dL), (curve A); home village at time of screening (villages with n<50 combined into ‘other’ giving 6 villages (curve B); year screened (curve C); age in weeks when screened (1–4 weeks), (curve D). Curves were censored at 18 months as there were very few lead-related outcomes after this and any link beyond 18 months, even environmental, to congenital levels was felt to be tenuous.

## Results

Between 1 July 2010 and 25 January 2018, 1508 children were identified with a BLC recorded at ≤1 month of age. Forty were excluded as they met the criteria for re-screening–outcomes are listed in Fig 1 for those individuals. The remaining 1468 children were included in the analysis, with outcomes dichotomised as ‘lead-related death or treatment’ (284, 19.3%) or ‘no lead related death or treatment’ (1184, 80.7%) (S1 Fig).

Table 1 shows the demographic and outcome characteristics of the included population with percentages rounded to 1 decimal place. The majority of those dichotomised as ‘lead-related death or treatment’ were patients who met criteria for treatment with DMSA (272, 95.8%), with 5 (1.8% of lead-related outcomes)) who presented with encephalopathy and were treated with EDTA, coded as EDTA, 5 and 7 (2.5% of lead-related outcomes) who met the above criteria for lead-related death. The ‘No lead-related treatment or death’ group is comprised of those with the outcome ‘discharged no chelation’ (938, 79.2% of ‘no lead-related’ outcome group), ‘on programme no chelation’ (213 (18.0%), ‘left programme no chelation’ (15, 1.3%), ‘died not lead related’ (prior to any lead related outcome) 18 (1.5%).

**Table 1:** Demographic and outcome characteristics.

Characteristic	No. of Children	% of Total
**Sex** *		
M	753	51.3%
F	715	48.7%
**Age in Weeks at Screening**		
1	208	14.2%
2	278	18.9%
3	643	43.8%
4	339	23.1%
**Village of Usual Residence**		
A	340	23.2%
B	604	41.1%
C	147	10.0%
D	24	1.6%
E	199	13.6%
F	120	8.2%
G	8	0.5%
I	23	1.6%
Other	3	0.2%
**Year Screened**		
2011	7	0.5%
2012	82	5.6%
2013	242	16.5%
2014	255	17.4%
2015	323	22.0%
2016	224	15.3%
2017	315	21.5%
2018	20	1.4%
**Outcome**		
Died Pb Related	7	0.5%
EDTA	5	0.3%
DMSA	272	18.5%
Died Not Pb Related	18	1.2%
Discharged No Chelation	938	63.9%
On Programme No Chelation	213	14.5%
Left Programme	15	1.0%

*Rounding to 1 decimal place for ease of reading resulted in some percentage totals being slightly < or > 100% (within 2%).

There was an even distribution between male/female and age in weeks at screening, with 64.3% of children usually residing in villages A and B and the remaining 35.7% distributed across villages C–I and other (from out of area, 3 children total).

All-cause mortality during the study period was 2.3%, 34 children, 7 of which met the criteria to be designated lead related and 27 of which met criteria to be coded not lead related. The 9 children who died during the study period who were not allocated a study outcome of death met the criteria for ‘died not lead related’ chronologically later than a lead-related outcome of DMSA or EDTA. Table 1 shows the outcome code used for analysis, S1 Table shows all 34 deaths with initial and final BLC and time to death from last BLC. Mean final BLC before death for those who met criteria for lead-related death was 40.6 μg/dL (SD 22.1 μg/dL) and for those who met criteria for non-lead related death before chelation was 25.4 μg/dL (SD 18.9 μg/dL).

**Fig 2:**
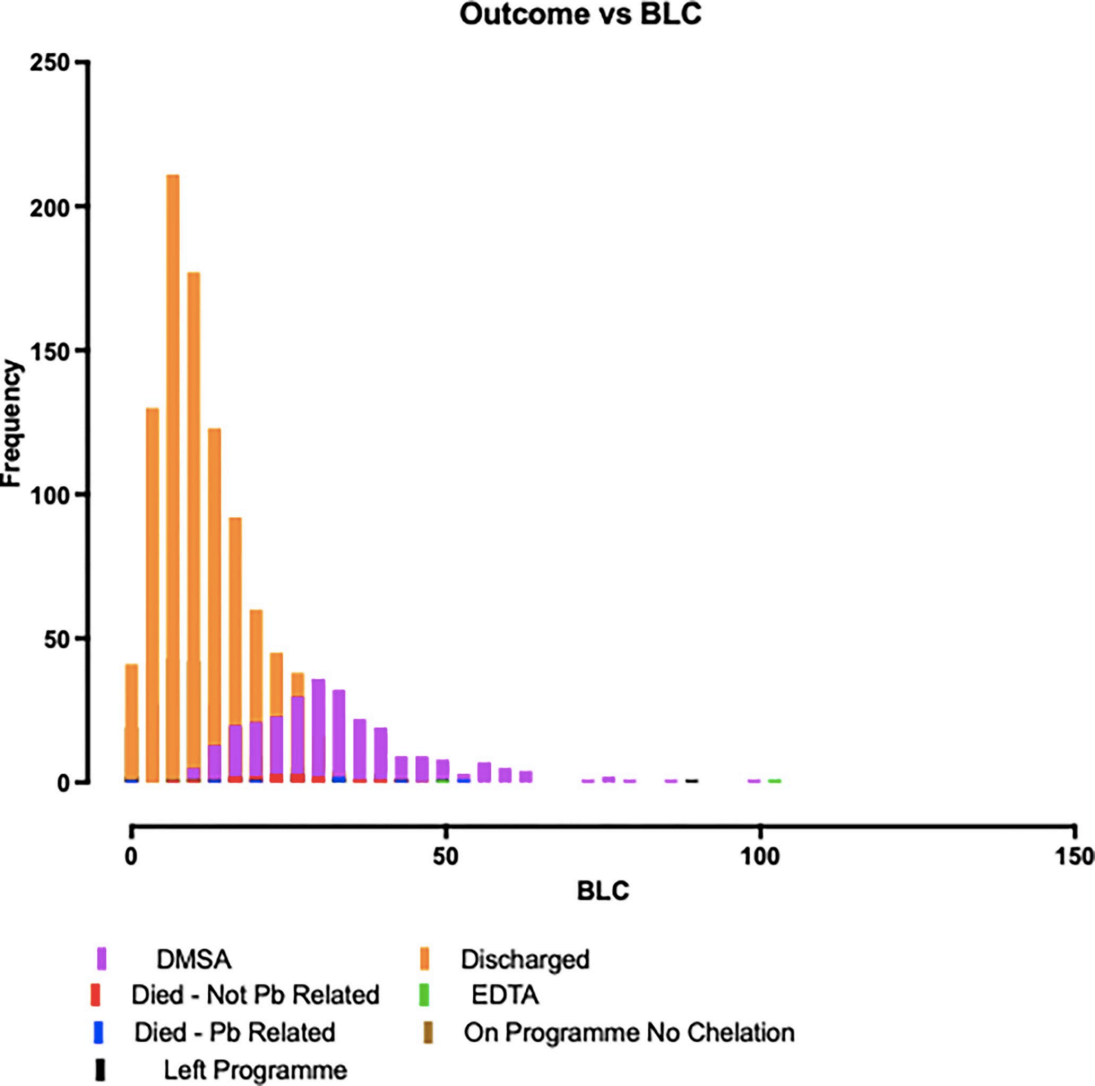
Stacked Bar Chart: First Ever BLC/Outcomes (Child).

Table 2 shows geometric mean initial neonatal BLC was 13.7μg/dL (geometric SD 2.00) and 56.2 μg/dL (geometric SD 1.52) amongst the small cohort of non-matched breastfeeding mothers tested. Fig 2 represents the frequency of outcome vs the BLC, with higher neonatal BLC’s more frequent with outcomes of lead-related death or treatment.

**Table 2:** Summary first ever blood lead concentrations (children and breastfeeding mothers).

**Summary Blood Lead Concentrations**	
**Children**	
Arithmetic Mean (child)	17.4 μg/dL (SD 12.5)
Geometric Mean (child)	13.7 μg/dL (GSD 2.00)
Median (child)	13.7 μg/dL
Range (child)	<3.3–105 μg/dL
**Breastfeeding Mothers**	
Arithmetic Mean (BFM)	61.4 μg/dL (SD 27.2)
Geometric Mean (BFM)	56.5 μg/dL (GSD 1.52)
Median (BFM)	56.6 μg/dL
Range (BFM)	18.1–154.8 μg/dL

**Fig 3:**
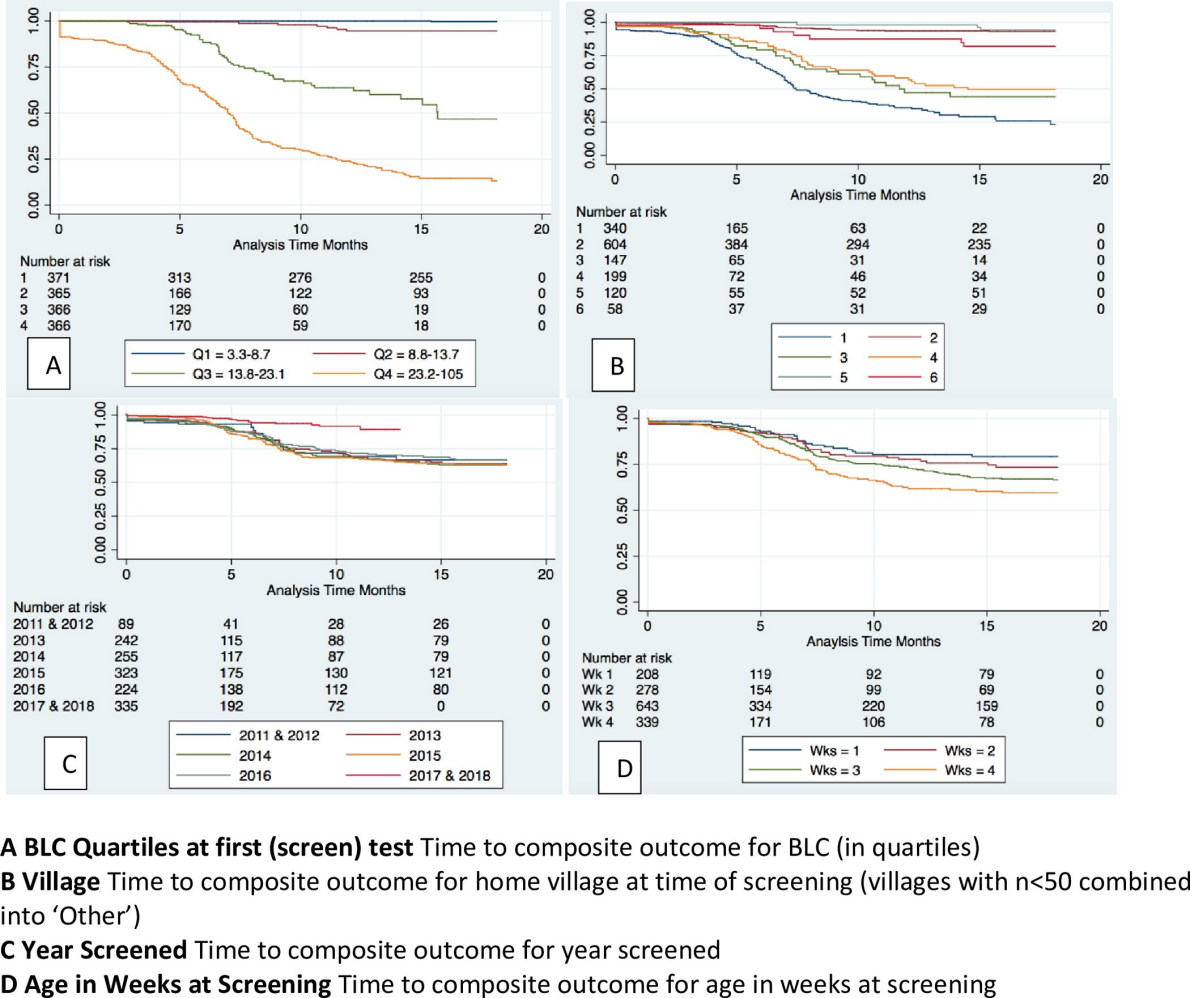
Kaplan Meier time-to-event curves for composite outcome (truncated at 18 months). **A. BLC Quartiles at first (screen) test** Time to composite outcome for BLC (in quartiles). **B. Village** Time to composite outcome for home village at time of screening (villages with n<50 combined into ‘Other’). **C. Year Screened** Time to composite outcome for year screened. **D. Age in Weeks at Screening** Time to composite outcome for age in weeks at screening.

Fig 3 shows time-to-event (TTE) analysis Kaplan-Meier curves for outcomes over time. In 3(A) children in the 4^th^ quartile of neonatal BLC (23.2–105 μg/dL) had earlier and more lead related outcomes than those with neonatal BLC in the lower 3 quartiles. A small proportion of this is due to those with neonatal BLC > = 45 μg/dL which confers an immediate lead-related outcome of being over the threshold for chelation. Children from villages known to have challenges with behavioural change for safer mining have a shorter TTE (Fig 3B) for lead-related outcomes. Year of screening had little role (3C), but age at time of neonatal BLC showed a shorter TTE for BLC taken in week 4 (3D).

**Fig 4:**
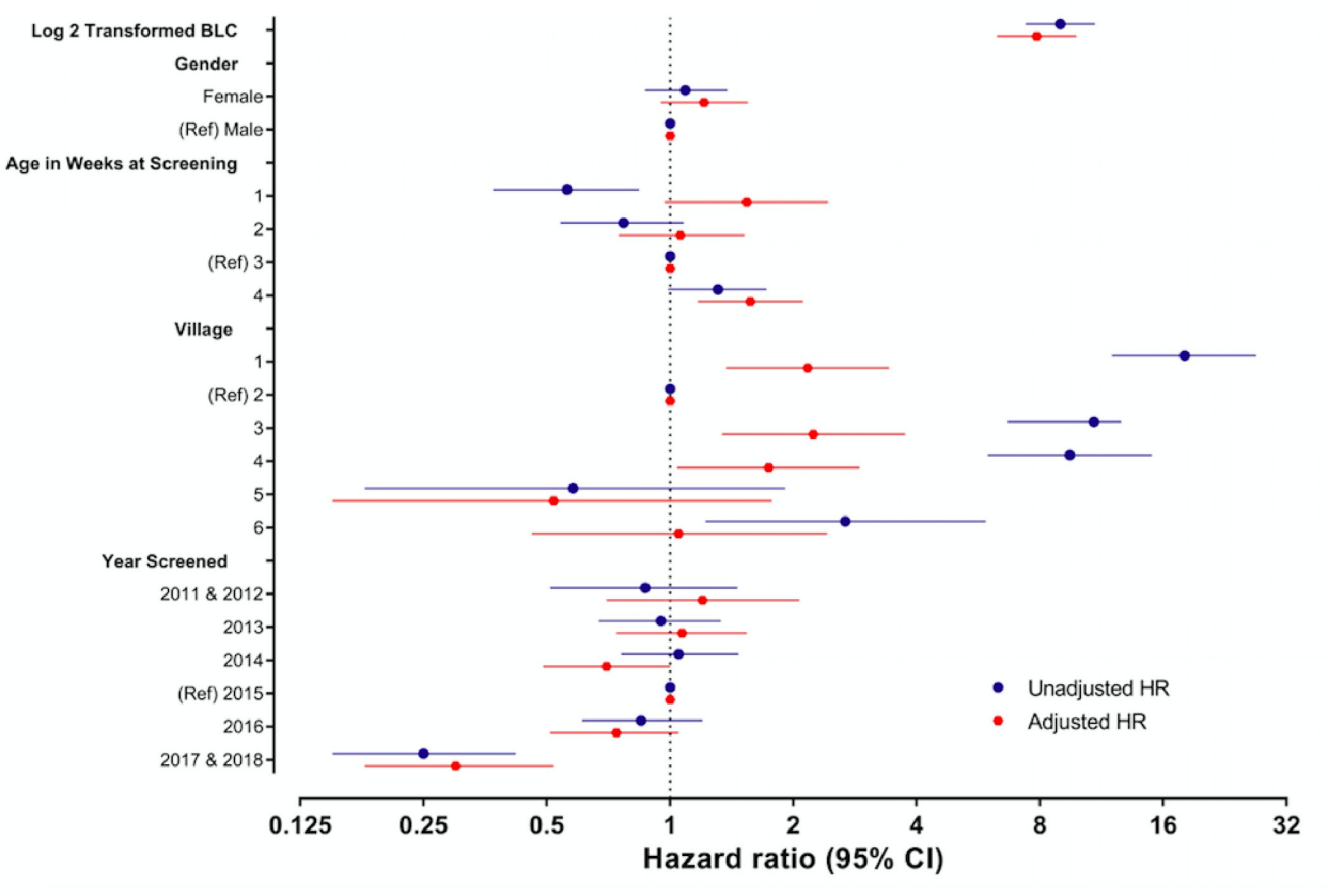
Forest plot of adjusted and unadjusted hazard ratios.

Table 3 outlines the Cox hazard ratios for the composite lead-related and non-lead related outcomes. For every doubling in BLC, the adjusted Cox hazard ratios showed an almost 8-fold increase in likelihood of a lead-related outcome (p<0.001) (Table 3; Fig 4). There was no significant difference in outcome between sex. Children who had their neonatal BLC taken in the 4^th^ week of life were 1.57 times more likely to have the composite lead-related outcome (p = 0.002), and village was also significantly associated with outcome, with those living in villages known to have issues with ongoing unsafe mining practices (per author experience and colleague reports rather than objective measure) statistically significantly more likely to have a lead-related outcome (p<0.001), particularly village 1 (adjusted HR 2.17 (1.37–3.43)). Neonates born in 2017–18 (the last year included) were significantly less likely to have a lead-related outcome (p<0.001) during the study period than those in previous years, with a trend downwards in hazard ratio since 2011 which did not reach statistical significance for the other years.

**Table 3:** Unadjusted and adjusted cox hazard ratios for composite lead-related outcome.

Characteristic	N	Unadjusted HR (95% CI)	Adjusted HR (95%CI)	p Value (adjusted model)
**Log 2 Transformed BLC**	**1468**	* **8.98 (7.39–10.90)**	**7.86 (6.29–9.82)**	**<0.001**
**Sex**				
Female	715	1.09 (0.87–1.38)	1.21 (0.95–1.55)	0.115
Male	753	Ref	Ref	
**Age in Weeks at Screening**				
1	208	0.56 (0.37–0.84)	1.54 (0.97–2.43)	0.066
2	278	0.77 (0.54–1.08)	1.06 (0.75–1.52)	0.731
3	643	Ref	Ref	
**4**	**339**	**1.31 (0.99–1.72)**	**1.57 (1.17–2.11)**	**0.002**
**Village**				
**1**	**340**	**18.06 (12.00–27.12)**	**2.17 (1.37–3.43)**	**0.001**
2	604	Ref	Ref	
**3**	**147**	**10.84 (6.67–12.64)**	**2.24 (1.34–3.75)**	**0.002**
**4**	**199**	**9.47 (5.97–15.03)**	**1.74 (1.04–2.90)**	**0.035**
5	120	0.58 (0.18–1.91)	0.52 (0.15–1.77)	0.298
6	58	2.68 (1.22–5.90)	1.05 (0.46–2.42)	0.905
**Year Screened**				
2011 & 2012	89	0.87 (0.51–1.46)	1.20 (0.70–2.07)	0.506
2013	242	0.95 (0.67–1.33)	1.07 (0.74–1.54)	0.715
2014	255	1.05 (0.76–1.47)	0.70 (0.49–1.00)	0.053
2015	323	Ref	Ref	
2016	224	0.85 (0.61–1.20)	0.74 (0.51–1.05)	0.095
**2017 & 2018**	**335**	**0.25 (0.15–0.42)**	**0.30 (0.18–0.52)**	**<0.001**

*Log 2 Transformation means that HR is per doubling of BLC (i.e. a 1 unit increase in log 2 BLC).

Fig 4 represents the hazard ratios for the composite outcome as a forest plot.

ROC curve analysis of 1468 observations gave an AUC of 0.92 (95% CI 0.90–0.93). Neonatal BLC at which there is 98% sensitivity for identifying children who subsequently have a lead-related outcome was ≥13.2 μg/dL (specificity 59%; positive likelihood ratio (+LR): 2.36). Neonatal BLC at which there is 95% sensitivity for identifying children who subsequently have a lead-related outcome is ≥15.0 μg/dL (specificity 67%; +LR 2.86).

## Discussion

We described the spectrum of congenital lead poisoning in an acute severe outbreak and analysed the association between neonatal BLC and medium-term lead-related outcomes during the study period.

To our knowledge, the Zamfara outbreak has resulted in the largest cohort of severely lead poisoned children ever reported. This analysis of time-to-lead-related outcomes in individuals with congenitally elevated BLC in Zamfara provide data to support decision making in responses to childhood lead poisoning outbreaks in low-or middle-income contexts where resource scarcity demands judicious allocation.

Given the reported high mortality overall associated with the outbreak prior to the initiation of the programme [8,9,11] (likely >400 fatalities), only 7 children screened as neonates and entered into the MSF programme had a death likely related to lead toxicity in the study period, 0.5% of the overall cohort, suggesting some impact from the response.

Geometric mean screening BLC was 13.7 μg/dL (geometric SD 12.5), with 92% of neonatal BLC being above the CDC level indicating action should occur (5 μg/dL) and 3.3% born with BLC above which chelation is recommended (>45 μg/dL). The maximum was 105 μg/dL, a level at which encephalopathy and death may occur. The geometric mean BLC of a cohort of breastfeeding mothers was 56.5 μg/dL (geometric SD 1.52). No matched data were available, however placental transmission and its impact is well documented [22]. No data are available regarding pre-term deliveries and miscarriage in this population.

Our hypothesis that children with higher neonatal BLC may be more at risk of post-partum exposure and therefore be more likely to have an outcome related to severe lead poisoning is supported by the finding that a key predictor of the lead-related composite outcome was neonatal BLC with an adjusted hazard ratio of 7.86 for the log 2 transformation meaning that for every doubling of BLC there was a 7.86-fold increase in the risk of a lead-related outcome within the study period. This suggests neonatal BLC is a predictor of environmental exposure over the first 18 months of life, with a high neonatal BLC indicating a continued risk of childhood exposure to a contaminated environment, even after remediation has occurred. Though all identified domestic and communal areas contaminated with lead were remediated [9], it took time for this to be completed, and for safer mining initiatives to begin in this complex context [15].

Village was a strong predictor of a lead-related outcome, with villages known by the authors to have more extensive artisanal mining activities and issues with source control and behavioural change being most strongly associated with adverse outcomes. The mean BLC from breastfeeding mothers underlines the importance of concurrently addressing exposure in women of childbearing age during an acute severe outbreak. The current recommendation from CDC is for mothers with BLC > = 40 μg/dL to cease breastfeeding until the BLC drops to <40 μg/dL, however given the likely minimal contribution of breast milk lead concentration to the child’s exposure, in this context with significant risks attached to formula feeding, breastfeeding was not discouraged. Both maternal and neonatal BLC are likely to indicate ongoing risks of environmental exposure.

In severe lead poisoning outbreaks in very low resource settings where judicious resource use is critical, responders could consider ceasing BLC monitoring in the context of multi-pronged population level exposure risk reduction if neonatal BLC is <13 μg/dL or <15 μg/dL. Only 2% and 5%, respectively, of those who progressed on to a lead related outcome had neonatal BLC below these cut-offs.

In Zamfara, despite a highly effective initial remediation programme in terms of soil lead concentration reduction [9], implementation of comprehensive safer mining initiatives occurred a few years after identification of the outbreak. Meaningful community engagement on environmental contamination was variable and behavioural change regarding prevention of ongoing childhood exposure was challenging. These neonatal BLC results over a period of 8 years likely reflect those challenges and underline the requirement for consistent, multipronged exposure mitigation early in severe lead poisoning outbreaks, however the hazard ratio for lead related outcomes trends down over the period studied, suggesting an overall decrease in exposure associated with the interventions.

## Limitations

A limitation of this study is that it utilises observational operationally collected BLC data with no matching environmental data. Furthermore, patients with outcome ‘discharged no chelation’ may have subsequently met chelation criteria due to lead exposure from re-contamination whilst no longer being monitored. This outcome means that beyond discharge what happened to the patient’s BLC is unknown as environmental conditions are not fully controlled. MSF is working in a complex security environment with very limited resources and therefore this pragmatic approach is required. It is not impossible but highly unlikely that some patients died from lead poisoning after being discharged–the team worked frequently in the communities and supported the local paediatric ward so would have become aware of most child deaths.

## Conclusion

Congenital lead poisoning predicts ongoing exposure in this large population of children with lead poisoning, even after environmental remediation. This suggests a complex, early, multidisciplinary approach to source control and exposure management is required when elevated neonatal BLC is observed in lead poisoning clusters in low-and middle-income contexts. Work is continuing to minimise exposure through safer mining initiatives, targeted remediation and chelation where indicated.

## Supporting information

S1 Fig Frequency distribution of lead-related and non-lead-related outcomes. (TIFF)

S2 Fig Box plot log 2 of BLC and week of age at screening. (TIFF)

S1 Table Mortality classification. (TIFF)

S1 Annex Zamfara chelation protocol. (PDF)
