# Correction: Neonatal blood lead concentration predicts medium term lead-related outcomes in children ≤5 years old with congenital lead poisoning: A retrospective cohort study in Northern Nigeria

**DOI:** 10.1371/journal.pgph.0002912

**Published:** 2024-02-01

**Authors:** Natalie Thurtle, Katharine A. Kirby, Jane Greig, Karla Bil, Paul I. Dargan, Godwin N. Ntadom, Nicholas A. Buckley

There are errors in the Funding and Competing Interests statements. The correct statements are:

**Funding:** Financial support for this research was absorbed by MSF budgets. MSF also provided support for this study in the form of salaries for NT, JG, and KB during part of the study period. The specific roles of these authors are articulated in the ‘author contributions’ section. MSF was involved in the study design, data collection and analysis, decision to publish, and preparation of the manuscript. Every stage of research and dissemination also received approval from the MSF (OCA) Research Committee.

**Competing Interests**: The authors have read the journal’s policy and have the following competing interests to declare: MSF provided support for this study in the form of salaries for NT, JG, and KB during part of the study period. This does not alter our adherence to *PLOS Global Public Health* policies on sharing data and materials. There are no patents, products in development or marketed products associated with this research to declare.
