# Supplementary figures and images for: Neonatal blood lead concentration predicts medium term lead-related outcomes in children ≤5 years old with congenital lead poisoning: A retrospective cohort study in Northern Nigeria
Source: PLOS Glob Public Health. 2023 Mar 29;3(3):e0001644. doi: 10.1371/journal.pgph.0001644 (PMC10057808; doi:10.1371/journal.pgph.0001644)

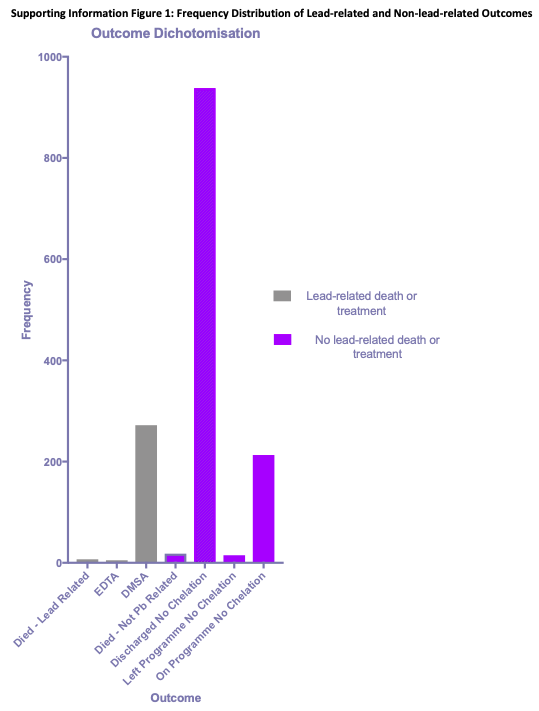

Supplement: S1 Fig — (TIFF) [file pgph.0001644.s001.tiff]

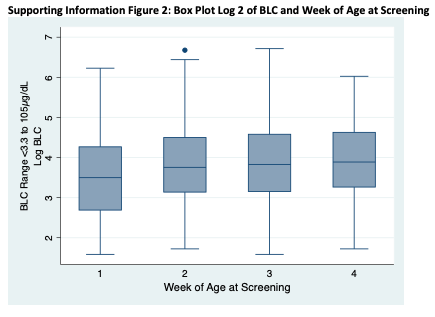

Supplement: S2 Fig — (TIFF) [file pgph.0001644.s002.tiff]

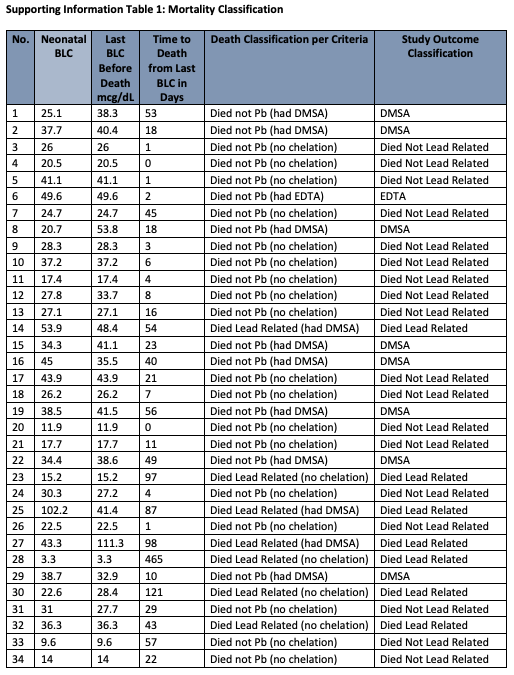

Supplement: S1 Table — (TIFF) [file pgph.0001644.s003.tiff]
