# Supplementary material for: Neonatal blood lead concentration predicts medium term lead-related outcomes in children ≤5 years old with congenital lead poisoning: A retrospective cohort study in Northern Nigeria
Source: PLOS Glob Public Health. 2023 Mar 29;3(3):e0001644. doi: 10.1371/journal.pgph.0001644 (PMC10057808; doi:10.1371/journal.pgph.0001644)
Supplement: S1 Annex — (PDF) [file pgph.0001644.s004.pdf]

Nigeria Lead Poisoning Protocol – May 2017 Revision
This protocol is for use only in children treated by Médecins Sans Frontières in Nigeria; it is not designed for other contexts

This protocol should be used together with the three page flowchart which gives an overview of the blood lead monitoring and DMSA chelation schedule for all patients.

Please refer to the 'Criteria for Severe Clinical Manifestations' box below. If the patient fulfils Criteria A discuss immediately with the Medical Team Leader (MTL).
The MedCo should be informed of all patients fulfilling Criteria A

If you are unsure about how you should manage your patient or have any other questions regarding patient management please discuss with the MTL.

Criteria for Severe Clinical Manifestations

It is important that a thorough clinical assessment is carried out in all children with a raised BLL in whom chelation therapy is planned. This is important in determining the chelation therapy of choice and the regimen that will be used. The criteria for severe clinical manifestations are listed in the box below.

CRITERIA FOR SEVERE CLINICAL MANIFESTATIONS

Any one of the following criteria:

Lead Related

A. ****Active convulsions, status epilepticus, altered consciousness level**
B. History of recent convulsion (last 2 days) and any two of the following criteria:
   - Wrist drop
   - Hypo or hyperreflexia
   - Hypo or hypertonia
   - Ataxia
   - Loss of Coordination
   - Other focal neurological signs with no other clinical explanation
   - Refusal of feeding, especially for breast feeding children
   - Loss of developmental milestones

Non-Lead Related

C. Signs of severe illness like malaria, meningitis, measles or severe pneumonia (admit to treat the life threatening disease and observe the patient).

******Patients fulfilling criteria A may be suitable for treatment with calcium disodium edetate (EDTA) rather than DMSA; see page 5 for further detail.

Re-Screening and Re-Enrollment
The following children who have been discharged from the lead poisoning programme should be rescreened and re-enrolled in the programme if they meet enrollment criteria (BLL >30 µg/dL):

Children Living in Compound Where *Another* Child Has Met Re-exposure Criteria
Children <4 yrs old ***living in a compound where another child has met re-exposure criteria*** (BLL ≥100 µg/dL **and/or** BLL rise of ≥50% between BLLs taken for any reason), regardless of whether the compound is found to be contaminated or not, should be re-screened.

Discharge Protocol
1) No child <2 years should be discharged from the programme from 1st April 2017. If a child meets discharge criteria based on BLLs they should have 3 monthly BLLs until 2 years of age at which point they can be discharged from the programme if they still meet discharge criteria AND they are not known to live in a grossly recontaminated compound or have a major unmitigated exposure pathway.

2) No child <4 yrs old should be discharged from the programme who is known to live in a grossly recontaminated compound until remediation has taken place.

3) No child <4 yrs old known to have a major unmitigated exposure pathway should be discharged from the programme until mitigation has been put in place.

<u>Chelation Treatment Criteria for patients with High VBLL</u>

Villages known to be remediated:

Chelation therapy is indicated in all children three years and under (if age unclear, use height < 112cm – 100th Centile for 4 yrs + 0 months) with a blood lead level (BLL) ≥ 45 µg/dL. In order to determine the therapy of choice and the regimen that will be used:

- First determine whether the patient fulfils Criteria A in the "criteria for severe manifestations" box on page 1.
  - If so, admission for Calcium disodium edetate (Ca2NaEDTA) therapy may be required – see pages 3-4 for further details.
  - If not, DMSA is the treatment of choice – refer to the flowchart pages 2-3 and see pages 5-6 of this document for further details.

Children living in compounds known to be re-contaminated with lead post-remediation:

Children living in compounds with *significant causative recontamination* (as defined by the environmental team) should be treated as per protocol but any child with BLL ≥100µg/dL should receive chelation DOT in Anka hospital and then should not return to the compound until it has been remediated. Health Promotion Team effort should go into facilitating this as the child is not able to advocate for themselves; suitable alternative places are compounds of family or friends in the same village that have been demonstrated to have no or minimal recontamination, or the hospital.

Villages known to be significantly contaminated but not remediated yet:

Treating patients with chelation therapy if they will return to exposure after completion of therapy or during therapy is largely a futile exercise. Therefore, only individuals with an immediate high risk of death or serious permanent morbidity (i.e. category A cases) will be treated with chelation therapy in villages that have not undergone large scale remediation (i.e. outside of the 8 villages where MSF has had previous or current Pb2+ OPD services).

It is important in category A patients being chelated from unremediated villages that all of the risks/ benefits are explained to their families. They should commit to protecting the patient from re-exposure – options include staying with family in non-contaminated villages, staying at the hospital, and as a last resort possible emergency interim remediation of individual compounds. As none of these options will be possible on large scale, and it cannot be guaranteed what families/caretakers will choose to do post-treatment despite pre-treatment promises, it is essential that strict criteria are followed. Admission of these patients has to be approved by the MTL and MedCo should be made aware.

<u>Hospitalisation policy:</u>

Patients from remediated villages that have regular outreach visits:

Patients ONLY need to be admitted if:

- They have one of the severe clinical manifestations of lead poisoning listed above, or;
- They have a significant co-morbidity

Otherwise DMSA therapy can commence in OPD outreach.

NB. EDTA is always IPD.

Patients from non remediated villages:

- Admission in the IPD for therapy with Ca2NaEDTA if they fit criteria ‘A’ above. If no Ca2NaEDTA is available, children who fit criteria A can be treated with DMSA via nasogastric tube using a 19-day DMSA course.
- Children from non-remediated villages should not be treated unless they fit Criteria ‘A’ above (i.e. are encephalopathic).

<u>CALCIUM DISODIUM EDETATE (EDTA) THERAPY</u>

This is indicated only in patients with severe active acute lead related encephalopathy:

1. Blood lead concentration ≥45μg/dL - usually these patients will have a blood lead level significantly greater than 120μg/dL.

AND

2. Criteria A in the "criteria for severe clinical manifestations" (see box on page 1)

Ca2NaEDTA therapy is given intravenously and can only be administered in the IPD and all cases should be discussed with the MTL.

Ca2NaEDTA should NOT be given to those with renal impairment without discussion with Dr Paul Dargan (or in his absence an alternate clinical toxicologist at the discretion of the Health Adviser) after discussion with the MTL. The MedCo should also be aware of all such patients.

Dosing schedule:
- Five day course of intravenous therapy
- 40 mg/kg dose to be given twice daily (total 80mg/kg per day)
- The Ca2NaEDTA should be diluted in 100-250mL sodium chloride (alternatively an equal volume of 5% dextrose can be used) and this solution should be given over at least an hour.

Other Considerations:
Check pre-EDTA baseline creatinine and perform a daily urine dipstick for proteinuria. If proteinuria is present, creatinine should also be checked daily. All patients should have creatinine checked on days 3 and 5. If there is a creatinine rise to >120micromol/L (or a rise of > 3x the baseline creatinine) Ca2NaEDTA should be stopped and the patient discussed with Dr Paul Dargan (or in his absence an alternate clinical toxicologist at the discretion of the Health Adviser) after discussion with the MTL.

Although it is not feasible to be undertaking strict fluid balance monitoring, it is important to check that children are passing urine. Place a plastic glove over the penis of male children and check this every 2 hours for urine. Document any urine passed on the vital signs chart in the space provided. For female children, ask the mother every 2 hours if the child has passed urine and document in the vital signs chart.

Over-hydration can potentially be risky in the context of significant cerebral oedema but it is important that patients should be kept well hydrated (euvolaemic) to decrease the risk of nephrotoxicity. If they are too drowsy to be taking oral fluids / breast feeding then supplementary intravenous or nasogastric fluids should be given.

If a child meeting criteria A has a Haemoglobin <7g/L, transfuse to ≥7g/L using standard MSF transfusion protocols. <u>The donor BLL should be less than the blood lead concentration of the child (ideally < 3.3mcg/dL) *unless the child has a life threatening anaemia and there is only one potential donor*</u>.

Procedure at the end of the 5 day Ca2NaEDTA course:
The child should have a blood taken for BLL, creatinine (and haemoglobin if this was abnormal on day 1 or if there is another clinical indication).

If the child still fulfils criteria A in the “severe clinical manifestations” box on page 1, then it is likely that another 5 day course of Ca2NaEDTA should be given immediately without a break. This should ONLY occur after discussion with Dr Paul Dargan (or in his absence an alternate clinical toxicologist at the discretion of the Health Adviser) following initial discussion with the MTL. The MedCo should also be aware of all such patients.

If the child no longer fulfils criteria A in the “severe clinical manifestations” box page on page 1:

a. If BLL ≥45µg/dL, continue **immediately** with DMSA as per the dosage schedule (1.1.) for first course DMSA on page 7 of this protocol (and on page 2 of the flowchart).

b. If BLL <45µg/dL, two weeks break from treatment, with repeat blood lead testing at 2 weeks. Interpretation of this blood lead should follow the confirmatory blood testing sheet on page 1 of the flowchart and if patients require DMSA chelation therapy this should be based on the dosage schedule (1.1) for first course DMSA below on page 7 of this protocol.

DMSA (SUCCIMER) THERAPY

DMSA therapy is indicated in patients (<4 years) from remediated villages who:

1. Have a blood lead concentration ≥ 45µg/dL
AND
2. *DON'T have* active, acute encephalopathy (i.e. they do not fulfil the Criteria A for "severe clinical manifestations" in the box on page 1)

Doses are based on 10mg/kg/dose

Weight in kg	<15**	15 – 24	25 - 34	35 – 44	≥ 45
Dose	100	200	300	400	500

****Dosing for Newborns <u><</u>5 kg** – this should be weight based (10mg/kg) and all newborns should be admitted to the IPD to ensure appropriate dose titration (as it is difficult to train mothers to split the capsules and ensure accurate dosing)

The DMSA protocols are summarised on pages 2 and 3 of the flowcharts. The first course of DMSA should *always* be a 19 day course.

FIRST COURSE OF DMSA:

All patients should have a Hb, creat and ALT on day 1. If ALT and creat are normal, continue with DMSA, if these are abnormal discuss with MTL.

1.1. DMSA dosing schedule:
Dose as above to be given 3x/day (tds) for 5 days followed by:

a. If Day 1 (pre-treatment) blood lead concentration ≥ 120 µg/dL continue 3x/day (tds) for the final 14 days

b. If Day 1 (pre-treatment) blood lead concentration 45-119 µg/dL decrease to 2x/day (bd) for the final 14 days

1.2. At end of DMSA therapy: All patients should have a venous blood lead level [1] and ALT (Hb and creatinine only required if abnormal on D1 or if there is another clinical indication).

a. If blood lead concentration **≥120 µg/dL,** then continue immediately with 2nd course of DMSA chelation therapy (using the dosing schedule (2.1.) below).

b. If blood lead concentration **80 – 119 µg/dL**, then no immediate DMSA, repeat venous blood lead concentration 2 weeks later and decision for further DMSA therapy using the flowchart (page 1 / 3) dosing schedule (2.1.) below.

c. If blood lead concentration **65 – 79 µg/dL**, then no immediate DMSA, repeat venous blood lead concentration 4 weeks later and decision for further DMSA therapy using the flowchart (page 1 / 3) dosing schedule (2.1.) below.

[1] To be used as an "end of course" test for further decision making the VBLL should be on at least day 17 of a 19 day treatment and no more than 10 days after the end the course. If it is later than this it is considered a follow-up test. If a patient absconded from the treatment course (see definition at the end of this document) and returns to the clinic, a blood sample can be taken for VBLL if it is at least 14 days since the last VBLL test, and it should be acted on as if a follow-up test.

d. If blood lead concentration **45 – 64 µg/dL**, then no immediate DMSA, repeat venous blood lead concentration 8 weeks later and decision for further DMSA therapy using the flowchart (page 1 / 3) dosing schedule (2.1.) below.
e. If blood lead concentration **<45 µg/dL**, then no immediate DMSA, repeat blood lead concentration according to the schedule on page 1 of the flowchart.

<u>SECOND AND SUBSEQUENT COURSE OF DMSA</u>:

2.1. DMSA dosing schedule:
Dose as above to be given 3x/day (tds) for 5 days followed by:
a. If Day 1 (pre-treatment) blood lead concentration **≥ 80 µg/dL** decrease to 2x/day (bd) for the final 14 days
b. If Day 1 (pre-treatment) blood lead concentration **45 – 79 µg/dL** stop DMSA therapy (i.e. only a total of 5 days therapy in these patients)

2.2. At end of DMSA therapy (D5 for 5 day courses and D19 for 19 day courses): All patients should have a venous blood lead level [2]and ALT (Hb and creatinine only required if abnormal on D1 or if there is another clinical indication).
a. If blood lead concentration **≥ 120 µg/dL***,* then continue immediately with 2nd course of DMSA chelation therapy (using the dosing schedule (2.1.) above).
b. If blood lead concentration **80 – 119 µg/dL**, then no immediate DMSA, repeat venous blood lead concentration 2 weeks later and decision for further DMSA therapy using the flowchart (page 1 / 3) dosing schedule (2.1.) above.
c. If blood lead concentration **65 – 79 µg/dL**, then no immediate DMSA, repeat venous blood lead concentration 4 weeks later and decision for further DMSA therapy using the flowchart (page 1 / 3) dosing schedule (2.1.) above.
d. If blood lead concentration **45 – 64 µg/dL**, then no immediate DMSA, repeat venous blood lead concentration 8 weeks later and decision for further DMSA therapy using the flowchart (page 1 / 3) dosing schedule (2.1.) above.
e. If blood lead concentration **<45 µg/dL**, then no immediate DMSA, repeat blood lead concentration according to the schedule on page 1 of the flowchart.

[2] To be used as an "end of course" test for further decision making the VBLL should be on at least day 17 of a 19 day treatment and no more than 10 days after the end the course, or on at least day 4 of a 5 day treatment and no more than 7 days after the end the course. If it is later than this it is considered a follow-up test. If a patient absconded from the treatment course (see definition at the end of this document) and returns to the clinic, a blood sample can be taken for VBLL if it is at least 14 days since the last VBLL test, and it should be acted on as if a follow-up test.

Annex 1: Supplements to be provided

All supplements are to be given throughout the chelating treatment course and 2 weeks after ceasing treatment.

VITAMIN C SUPPLEMENT

	Using 500mg tabs:
Children	½ tab bd

CALCIUM SUPPLEMENT

	Using 500mg calcium carbonate tabs:
< 1yr	0.5 tab at midday
1 – 3 yrs	1 tab at midday
4 – 8 yrs	1.5 tabs at midday

MULTIVITAMIN SUPPLEMENT

Children	1 tab at midday

ZINC SUPPLEMENT

	Using 20mg zinc sulphate tabs:
< 10kg	¼ tab at midday
10 – 25 kg	½ tab at midday
> 25kg	1 tab at midday

IRON SUPPLEMENT

Dosage is dependent on weight and Hb.

Hb > 12	Using ferrous sulphate 60mg and folic acid 0.4 mg:
<16kg	¼ tab at midday
16 – 30kg	½ tab at midday
>30kg	1 tab at midday

Hb 11 – 12	Using ferrous sulphate 60mg and folic acid 0.4 mg:
<16kg	1/2 tab at midday
16 – 20kg	1 tab at midday
>20kg	1 ½ tabs at midday

Hb < 11	Using ferrous sulphate 60mg and folic acid 0.4 mg:
Dose halved for the first 2 days	
<12kg	½ tab at midday
12 – 20kg	1 tab at midday
>20kg	1 ½ tabs at midday

ANNEX 2:

DEFAULTER TRACING FLOW CHART

MA= Missed Appointment
HP= Health Promotion

FIRST MISSED APPOINTMENT

- DAY 19 BLOOD
- F/U BLOOD
- TREATMENT DAY
- ACTION DUE DAY

NEXT COMMUNITY DAY/VISIT: CHEWs will trace child and offer 1st counseling with agreed messages

NEXT CLINIC VISIT: Town crier to remind Guardian to bring child to the clinic.

1WEEK AFTER FIRST MA: Trace child and offer second counseling with agreed messages.

3 WEEKS AFTER 1ST MA: CHEWs + HP to trace child/DEFAULTER and offer third counseling (individual/group counseling; yet to be agreed upon). Commence defaulter tracing documentation on Defaulter Tracing Form.

7 WEEKS AFTER 1st MA: Trace child and offer fourth counseling + agreed messages.

12 WEEKS (3 MONTHS) AFTER FIRST MA: Offer 5th and final counseling.

13th WEEK: If they do not return within one week, they are designated as “***LEFT PROGRAMME***”.

THEY MAY RETURN TO THE PROGRAMME, BUT OTHERWISE WILL NOT BE ACTIVELY FOLLOWED UP AFTER THIS POINT.

ANNEX 3: DEFINITIONS:

- Screened:
  Any patient from whom we have ever taken a BLL test.

- Enrolled (for follow-up):
  Any patient who has a screening blood lead level $\geq$ 30 mcg/dL. (Formerly any patient who had a screening blood lead level ≥10µg/dL) If they leave the programme or are discharged they are no longer enrolled but they once were, so should be included in 'total patients ever enrolled'.

- Admitted (for treatment):
  Started DMSA/Ca2NaEDTA course at any point.

- Discharged (from the programme):
- Children > 2 years of age who are not known to live in a recontaminated compound or have another unmitigated exposure source who have a single blood lead level <30µg/dL, or a BLL <45µg/dL on three separate consecutive blood lead samples over a period of at least 4 months (tests at least 2 months apart).
- (Note, prior to October 2011, the discharge criteria was a single BLL <10µg/dL, and from October 2011 until July 2016 it was a blood lead level <30µg/dL on two blood samples at least 3 months apart).
- The discharge criterion for children currently ≥4 years old is a single BLL <45µg/dL.

- Absconded from course:
  A patient who has missed more than 7 treatment days in a 19 day DMSA course.
  This is the outcome for that course (course not completed) and it does not preclude the patient being given further courses as needed.

- Absconded from VBLL test:
  End of course test: A patient who has missed an end of course test by more than 10 days at the end of a 19 day DMSA course, or more than 7 days at the end of a 5 day course.

- Hospitalisation:
  Patients are admitted to the hospital IPD.

- Left programme:
  A patient may be given an outcome of left programme for various reasons, such as:
  - has been absent from the programme for more than 13 weeks as per defaulter tracing flow chart.
  - is known to have moved permanently away from the catchment area.
